# Staphylococcal Toxic Shock Syndrome Caused by Tampon Use

**DOI:** 10.1155/2015/640373

**Published:** 2015-01-26

**Authors:** Cian McDermott, Michael Sheridan

**Affiliations:** Emergency Department, University Hospital Geelong, Ryrie Street, Geelong, VIC 3220, Australia

## Abstract

The authors report a case of near-fatal sepsis with multiorgan failure resulting from a *Staphylococcal* tampon-associated toxic shock syndrome, requiring a lengthy critical care admission. Successful treatment of this condition focuses on early identification, source control, and administration of antimicrobial agents. Intravenous immunoglobulin therapy used early may prevent widespread tissue necrosis.

## 1. Case Report

A 15-year-old Caucasian schoolgirl presented to the Emergency Department (ED) complaining of a single day history of lower abdominal pain, muscle aches, diarrhoea, and vomiting. She had a tampon in situ for 24 hours for menstrual bleeding. She had been undergoing treatment for thyroid nodular disease using carbimazole 10 mg twice daily, which she had ceased 3 days previously.

On initial examination, the patient was hypotensive (systolic blood pressure 75 mmHg), pyrexic (temperature 39.4°C), and tachycardic (heart rate 150/minute). There were signs of multiorgan dysfunction as her skin peripheries were profoundly vasoconstricted and mottled with a significant delay in capillary refill time (10 seconds) and an elevated serum lactate (13.0 mmol/L). The patient was acutely confused and intermittently drowsy. There was a generalized lower abdominal tenderness. Vaginal examination revealed a malodorous tampon, coated in a green mucopurulent discharge, which was removed.

The patient was sedated, intubated, and ventilated in the ED. Vasopressor support, aggressive fluid resuscitation in addition to broad-spectrum, empirical antibiotics (vancomycin, flucloxacillin, piptazobactam, and clindamycin) for sepsis of unknown origin and intravenous immunoglobulin treatment, was commenced. Initial investigations showed a white cell count of 17.4 × 10^9^/L, neutrophil count of 16.4 × 10^9^/L, CRP of 206 mg/L, and a venous serum pH of 7.03, with a base excess of −18.5 mmol/L and a bicarbonate level of 12 mmol/L. High vaginal swabs returned a heavy growth of methicillin-susceptible* Staphylococcal aureus*. Toxin gene analysis subsequently confirmed the presence of the superantigen exotoxin, toxic shock syndrome toxin-1 (TSST-1).

In the intensive care unit, her management was complicated by an acute kidney injury requiring renal replacement therapy, a sepsis-associated coagulopathy, septic encephalopathy, transient cardiomyopathy, and thyrotoxicosis. Adult respiratory distress syndrome and a* Pseudomonas aeruginosa* pneumonia further complicated this patient's recovery. Following two failed extubation attempts and difficulty to wean from ventilatory support, a tracheostomy was sited. This patient was eventually decannulated and discharged to the paediatric ward following a 1-month critical care admission.

## 2. Discussion

Toxic shock syndrome (TSS) is an acute, multisystem, toxin-mediated condition often preceded by a prodromal influenza-like illness, leading to rapid-onset shock, erythroderma, and accelerated multiorgan failure.

*Staphylococcal* TSS was first described among a cohort of young women inthe USA in the 1980s and was associated with the use of super-absorbent tampon material [1].

Bacterial toxins act as highly virulent superantigens that trigger massive immune cell activation and cytokine release resulting in distributive shock and multiorgan failure. In 95% of menstrual cases of TSS,* staphylococcal* TSST-1 is the causative agent [2]. Host antibody deficiency is also recognised as an important factor in the development of fulminant TSS [2, 3].

Therapeutic strategies include early aggressive resuscitation, supportive management, and source control [2, 4, 5]. Surgical debridement may be necessary if tissue infection is established. Vaginal examination and foreign body removal should be considered in female patients. Antibiotic therapy should focus on reducing bacterial load and exotoxin production. Specific antimicrobial therapies such as clindamycin or linezolid (these neutralize TSST1 production) and intravenous immunoglobulin have been shown to improve survival in TSS and their early use is recommended [4, 5].

Despite changes in the design of female sanitary products and a consequent decline in the incidence of* staphylococcal* TSS, cases of menses-associated TSS still occur [2]. Emergency physicians should maintain a high index of suspicion in all women using catamenial products that present with these symptoms. Recurrent TSS has been reported and these women should be advised against future use of tampons.
